# THE MODERATING ROLE OF BIRTHPLACE ON DENTAL CLEANING SERVICE AMONG CHINESE OLDER ADULTS IN HAWAII

**DOI:** 10.1093/geroni/igz038.2260

**Published:** 2019-11-08

**Authors:** Sizhe Liu, Wei Zhang, Keqing Zhang, Bei Wu

**Affiliations:** 1 University of Hawaii at Mānoa, Honolulu, Hawaii, United States; 2 New York University, New York, New York, United States

## Abstract

Regular dental cleaning is vital to maintaining good oral health. This study aims to identify socio-demographic characteristics that are associated with the use of dental cleaning services among Chinese older adults in Honolulu, Hawai’i. In addition, we examine if birth-place moderates these associations. The data for this study were collected from 398 Chinese older adults living in Honolulu. Results from multivariate logistic regressions showed that those who were married and with higher levels of education were more likely to have their teeth cleaned within the past year compared to those who were not married or with lower levels of education. These significant associations were only found salient for the foreign-born when the moderating role of birth place was accounted for in the model. These differences may be partially due to the impact of acculturation and the knowledge of oral health and dental services.

